# Supplementary material for: Central neural circuitry mediating courtship song perception in male Drosophila
Source: eLife. 2015 Sep 21;4:e08477. doi: 10.7554/eLife.08477 (PMC4575990; doi:10.7554/eLife.08477)
Supplement: Figure 1—source data 1. — DOI: http://dx.doi.org/10.7554/eLife.08477.004 [file elife08477s001.docx]

|  | Genotype | Male | Female |
| --- | --- | --- | --- |
| aPN1 | *LexAop2-FLP/+; fru^LexA^ , UAS>stop>myr::GFP/21B12-GAL4* | 4.6 ± 0.3 | 4.8 ± 0.5 |
|  | *LexAop2-FLP/+; fru^LexA^ , UAS>stop>myr::GFP/22B11-GAL4* | 4.7 ± 0.4 | 5.2 ± 0.5 |
|  | *LexAop2-FLP/+; fru^LexA^ , UAS>stop>myr::GFP/49F09-GAL4* | 4 ± 0.3 | 3.7 ± 0.4 |
| VPN1 | *LexAop2-FLP/+; fru^LexA^ , UAS>stop>myr::GFP/46F09-GAL4* | *3.2 ± 0.3 | 0 ± 0 |
|  | *LexAop2-FLP/+; fru^LexA^ , UAS>stop>myr::GFP/72E10-GAL4* | *4.5 ± 0.3 | 0 ± 0 |

Figure 1-source data 1. Quantification of aPN1 or VPN1 neurons labeled by intersectional drivers

Cell numbers of aPN1 or VPN1 neurons per hemisphere were quantified. Values are mean ± SEM. n=6~10 for all the genotypes. *p<0.001 compared to cell numbers in females (Student t test).
